# Neuronal activity (c-Fos) delineating interactions of the cerebral cortex and basal ganglia

**DOI:** 10.3389/fnana.2014.00013

**Published:** 2014-03-26

**Authors:** Mei-Hong Qiu, Michael C. Chen, Zhi-Li Huang, Jun Lu

**Affiliations:** ^1^State Key Laboratory of Medical Neurobiology and Department of Neurobiology, School of Basic Medical Science, Fudan UniversityShanghai, China; ^2^Neurology Department and Division of Sleep Medicine, Beth Israel Deaconess Medical Center and Harvard Medical SchoolBoston, MA, USA; ^3^Department of Pharmacology, School of Basic Medical Science, Fudan UniversityShanghai, China; ^4^Institute of Brain Science, Fudan UniversityShanghai, China

**Keywords:** cerebral cortex, basal ganglia, arousal, atropine, 6-hydroxydopamine, c-Fos, rat

## Abstract

The cerebral cortex and basal ganglia (BG) form a neural circuit that is disrupted in disorders such as Parkinson’s disease. We found that neuronal activity (c-Fos) in the BG followed cortical activity, i.e., high in arousal state and low in sleep state. To determine if cortical activity is necessary for BG activity, we administered atropine to rats to induce a dissociative state resulting in slow-wave electroencephalography but hyperactive motor behaviors. Atropine blocked c-Fos expression in the cortex and BG, despite high c-Fos expression in the sub-cortical arousal neuronal groups and thalamus, indicating that cortical activity is required for BG activation. To identify which glutamate receptors in the BG that mediate cortical inputs, we injected ketamine [*N*-methyl-d-aspartate (NMDA) receptor antagonist] and 6-cyano-nitroquinoxaline-2, 3-dione (CNQX, a non-NMDA receptor antagonist). Systemic ketamine and CNQX administration revealed that NMDA receptors mediated subthalamic nucleus (STN) input to internal globus pallidus (GPi) and substantia nigra pars reticulata (SNr), while non-NMDA receptor mediated cortical input to the STN. Both types of glutamate receptors were involved in mediating cortical input to the striatum. Dorsal striatal (caudoputamen, CPu) dopamine depletion by 6-hydroxydopamine resulted in reduced activity of the CPu, globus pallidus externa (GPe), and STN but increased activity of the GPi, SNr, and putative layer V neurons in the motor cortex. Our results reveal that the cortical activity is necessary for BG activity and clarifies the pathways and properties of the BG-cortical network and their putative role in the pathophysiology of BG disorders.

## INTRODUCTION

The cerebral cortex and basal ganglia (BG), two of the largest forebrain structures, form a dynamic interactive network. The cerebral cortex, along with the thalamus and subcortical systems, sends signals to the BG. The BG in turn, with modulation from midbrain dopaminergic system, integrates and processes this cortical information for output, back to the cerebral cortex to shape cortical activity and ultimately affect cortical functions such as motor behavior (Yelnik, 2008). This reciprocal regulation is significantly altered after BG dysfunction, as in neurological disorders such as Parkinson’s disease and Huntington’s disease. Given the complex interconnections within the subunits of the BG, and between the BG and cerebral cortex, it is critical to clarify how the cerebral cortex and BG interact and how this interaction is shaped by subcortical modulation, especially by dopamine.

Classically, BG and cerebral cortex networks have been organized into direct, indirect, and hyper-direct pathways. Dopamine D_1_ and D_2_ receptor containing medium spiny neurons in the striatum (dorsal part, or caudate-putamen, CPu) receive glutamatergic cortical input and project to the internal segment of the globus pallidus (GPi)/substantia nigra pars reticulata (SNr; direct pathway) and external segment of globus pallidus (GPe; indirect pathway). The GPe and SNr/GPi have GABAergic projections to the mediodorsal thalamus (MD) and the motor thalamus, respectively (Nauta, 1979; Parent and De Bellefeuille, 1983; Haber et al., 1985), which innervate the medial prefrontal, motor, and somatosensory cortices, respectively. In addition, the glutamatergic subthalamic nucleus (STN) also receives cortical inputs (hyper-direct pathway) and in turn projects primarily to the GPe, GPi, and SNr. Midbrain dopaminergic neurons provide the major subcortical input to the BG, especially the striatum. These projections form the core of the classical model of BG organization that influences current thinking about BG function and dysfunction, as in Parkinson’s and Huntington’s disease.

Single-unit recordings show that neurons in the BG, i.e., the striatum, GPe, SNr, and STN, are more active during wake and rapid eye movement (REM) sleep and less active and synchronized to cortex electroencephalography (EEG) in slow-wave sleep or in an anesthetized state (Detari et al., 1987; Magill et al., 2000; Urbain et al., 2000; Mahon et al., 2006). Consistent with this evidence, we have previously demonstrated that c-Fos expression (a marker of neuronal activity) in BG subunits is higher during spontaneous wake and methamphetamine-induced arousal, than during sleep (Vetrivelan et al., 2010). These studies suggest that excitatory cortical input to the BG may regulate BG activity. Given that the BG also receives subcortical inputs and projects to the cortex, it is unclear whether the cortical activity is necessary for BG activity.

We examined BG-cortex activity using c-Fos expression after wakefulness, systemic atropine injection, and after blocking NMDA and non-NMDA receptors. Our results suggest that cortical neuronal activity is necessary for BG neuronal activity and that the cortex shapes BG activity via two pathways: NMDA/non-NMDA receptor-mediated cortical inputs to the striatum, and non-NMDA receptor-mediated cortical inputs to the STN. We then examined if cortical activity is affected by BG activity alterations caused by dopamine depletion. Striatal dopamine depletion resulted in hyperactivity of the GPi, SNr, and motor cortex, as well as hypoactivity of the dorsal striatum, GPe, and STN, but only during high cortical activity wakefulness. These changes in BG and cortical activity during dopamine depletion may underlie the various motor and non-motor symptoms of BG disorders, including Parkinson’s disease.

## MATERIALS AND METHODS

### ANIMALS

Pathogen-free, adult, male Sprague–Dawley rats (275–300 g, Harlan) were individually housed and had *ad libitum* access to food and water. All animals were housed under light-controlled conditions (12 h light starting at 07:00 AM, 100 lux) in an isolated ventilated chamber maintained at 20–22°C, All protocols were approved by the Institutional Animal Care and Use Committees of Beth Israel Deaconess Medical Center and the experiments were carried out in accordance with the Guidelines laid down by the NIH in the US regarding the care and use of animals for experimental procedures. Every effort was made to minimize the number of animals used and any pain and discomfort experienced by the subjects.

### ACTIVE-WAKE (AW) CONDITION

Four cages were aligned without covers in a hood, after saline or drug injection (see below), rats were allowed to freely move over and between cages. Under this social and motor-active condition, rats will remain awake and highly active for at least 4 h, as previously reported (Gompf et al., 2010). Rats were perfused 2 h after AW condition. The sleep control group consisted of animals that were perfused 2 h after saline injections around 9:00–10:00 AM. The perfused brains were processed for histology.

### 6-HYDROXYDOPAMINE (6-OHDA) INJECTIONS

Animals were anesthetized with chloral hydrate (350mg/kg) and received unilateral 6-OHDA (6%, 90 nl, Sigma) injections into the ventral GPe (coordinates: AP = 0.8 mm, ML = 3.0 mm, DV = 7.0 mm) through a glass pipette by air pressure. Animals were allowed to recover for 2 weeks prior to experiments and perfusion. This method of dopamine depletion has been confirmed in previous reports (Abedi et al., 2013).

### DRUG ADMINISTRATION SCIENCE AND ENGINEERING REFERENCES

Atropine (100 mg/kg, Sigma), Ketamine (35 mg/kg, Sigma), and CNQX (3.0 mg/kg, Sigma) were dissolved in saline immediately before use and administered via intraperitoneal injection between 9:00 and 10:00 AM.

### EEG/EMG RECORDING

We recorded EEG/EMG and video in two rats received atropine injections for 2 h, to confirm that atropine produces slow wave EEG and locomotion with high EMG. The surgery procedure and EEG analyses have been described in many of our previous publications (Lu et al., 2006; Vetrivelan et al., 2009; Qiu et al., 2010).

### IMMUNOHISTOCHEMISTRY

Animals were deeply anesthetized by chloral hydrate (500 mg/kg) and perfused with 50 ml saline followed by 500 ml 10% formalin through the heart. The brains were removed, post-fixed for 4 h in 10% formalin, and equilibrated in 20% sucrose in phosphate-buffered saline (PBS) overnight. The brains were frozen and sectioned on a freezing microtome at 40 μm into four series. Sections were washed in 0.1 M PBS, pH 7.4 (two changes) and then incubated in the primary antiserum (c-Fos, 1:50 K, AB5, Oncogene; choline acetyltransferase, ChAT, 1:500, AB144, Chemicon; tyrosine hydroxylase, TH, 1:20K, #22941, Diasorin) for 24 h at room temperature. On the second day, the sections were washed in PBS and incubated in biotinylated secondary antiserum (against appropriate species IgG, 1:1,000 in PBS) for 1 h, followed by a 1:1000 dilution of avidin-biotin-peroxidase (Vector Laboratories, Burlingame, CA, USA) for 1 h at room temperature. The peroxidase reaction was visualized with 0.05% 3,3-diaminobenzidine tetrahydrochloride (DAB, Sigma) in PBS and 0.01% H_2_O_2_. The sections were stained brown with DAB only or black by adding 0.05% cobalt chloride and 0.01% nickel ammonium sulfate to the DAB solutions. c-Fos was labeled black and TH and ChAT labeled brown to enable distinct staining within the same section. After terminating the reaction by PBS-azide, sections were mounted, dehydrated and cover slipped. As controls, adjacent sections were incubated without the primary antibody to confirm that no non-specific staining had occurred.

### CELL COUNTING

c-Fos positive neurons were counted in the BG of three adjacent sections that contain BG structures. For the striatum, the 2.0 mm × 2.0 mm counting box was placed in the center of the dorsomedial striatum. For c-Fos counting in M1/M2 cortex, only black-stained large neurons (likely projecting pyramidal neurons) were counted in the entire M1/M2 cortex. For other BG subunits, we counted all c-Fos positive neurons in the entire region of three adjacent sections. The c-Fos counts represented by average counting per section per side were used to construct **Tables 1** and **2**. We used, paired *T*-test for count differences.

**Table 1:** c-Fos expression in BG and cortex.

	Sleep	AW	Atropine	Ketamine	CNQX
Striatum	15.6 ± 7.0	179.0 ± 15.2	23.5 ± 5.7**	75.7 ± 13.4*^/##^	134.3 ± 11.4*^/##^
GPe	5.8 ± 7.2	60.0 ± 7.9	8.4 ± 2.6*	25.0 ± 4.1*^/##^	8.7 ± 2.3**^/#^
GPi	2.2 ± 0.9	24.2 ± 5.5	2.0 ± 0.7**	4.2 ± 1.3**	3.43 ± 0.9**
STN	2.2 ± 1.6	97.8 ± 9.0	10.6 ± 3.2**^/#^	85.2 ± 8.2^##^	6.2 ± 1.9**
SNr	1.8 ± 1.3	23.6 ± 4.3	1.8 ± 1.4**	1.9 ± 1.3**	2.2 ± 1.9**
LC	–	++++	++++	++++	+++
Cortex	Inactive	Active	Inactive	Active	Active
Thalamus	Inactive	Active	Active	Active	Active

LC: “+” = 25 c-Fos positive neurons. All c-Fos counting is presented by number/section/side. The difference *p < 0.05, **p < 0.01 of *T*-test is compared to the AW. The difference of #*p* < 0.05, ##*p* < 0.01 is compared to sleep condition.

**Table 2:** c-Fos expression in intact (DA+/+) vs. DA depleted side (DA-/-) in AW *p < 0.05, **p < 0.01.

	DA+/+	DA-/-
Striatum	185.3 ± 16.2	95.4 ± 20.2*
GPe	55.2 ± 8.8	32.5 ± 13.1*
GPi	22.5 ± 6.8	75.9 ± 19.2**
STN	105.8 ± 12.6	64.7 ± 8.5*
SNr	26.1 ± 5.5	75.3 ± 12.2**
M1–M2	15.0 ± 10.1	38.7 ± 15.8**

## RESULTS

### CORTICAL ACTIVITY PREDICTS BG ACTIVITY

Although we have previously shown c-Fos expression in the BG during spontaneous and methamphetamine-induced wake (Vetrivelan et al., 2010), we sought to determine c-Fos expression in the BG during an active-wake (AW) condition, in which rats remain spontaneous active awake in the sleep period (8:00 AM–12:00 PM) and engage in social and motor behavior (see Materials and Methods). To determine BG activity in the AW condition, we injected saline and perfused rats in two conditions: after 2 h of sleep (*N* = 5) or after 2 h of AW (*N* = 5), during which animals had high locomotion and social interactions. Animals were perfused between 11:00 AM and 12:00 PM. The perfused brains were sectioned at 40 μm in four series, and two series of sections each were double immunolabeled with c-Fos (black color) and TH (brown color), or c-Fos (black color) and ChAT (brown color), respectively.

**FIGURE 1:**
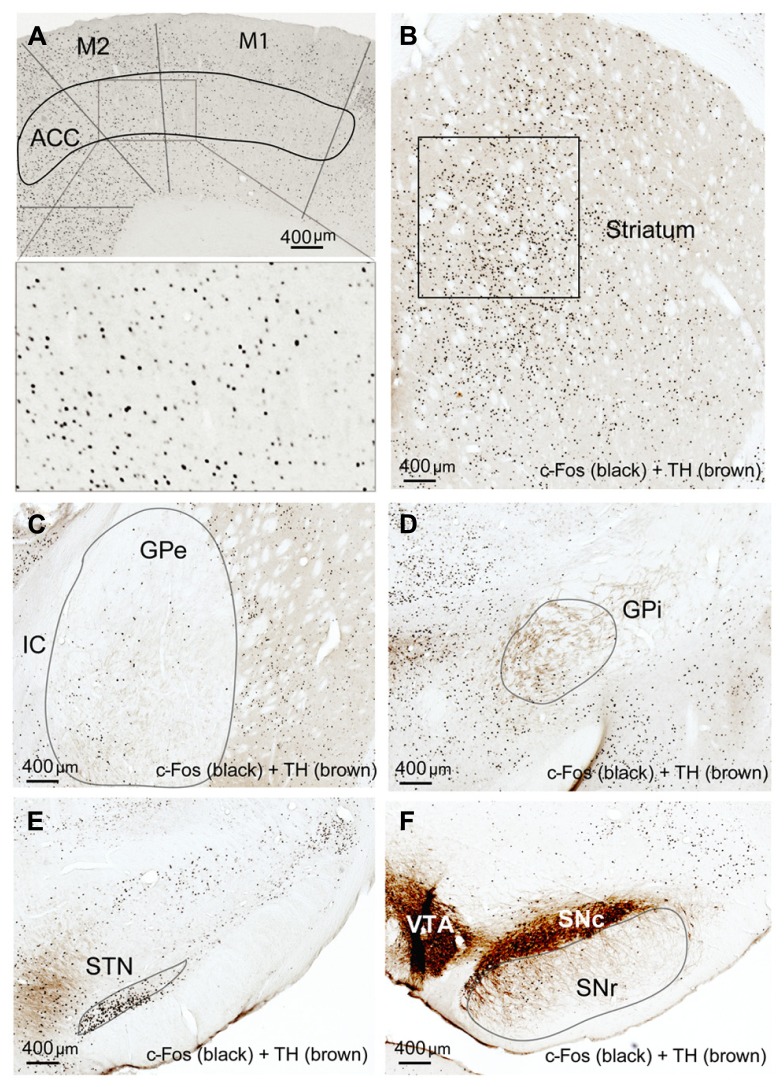
**Active-wake (AW) induces c-Fos expression in BG and cortex.** High c-Fos expression is seen in the cerebral cortex **(A)**, striatum **(B)**, GPe **(C)**, GPi **(D)**, STN **(E)**, and SNr **(F)** by AW condition. c-Fos active neurons are clustered in the medial part of the dorsal striatum, GPe, GPi, and SNr, which have strong interconnectivity with motor cortex.

Compared to the sleep condition, c-Fos expression was significantly higher in the cerebral cortex in AW rats, especially in the prefrontal and frontal, motor, and sensory cortices (**Table 1**; **Figure 1A**) and in all the subunits of the BG, i.e., the dorsal striatum (i.e., caudoputamen, CPu), GPe, GPi, STN, and SNr (**Table 1**; **Figures 1B–F**). There was a distinct pattern of c-Fos expression in the dorsomedial part in the striatum that receives inputs from the premotor (M2) and primary motor (M1) cortices (**Figure 1B**). The medial part of the GPe, GPi, SNr, and STN also showed strong c-Fos expression (**Table 1**; **Figures 1C–F**). The pattern and amount of waking c-Fos expression were similar to that following methamphetamine injection but much higher than spontaneous wakefulness (Vetrivelan et al., 2010). These results indicate that BG neural activity is increased when cortical neuronal activity is increased and likewise decreased when cortical activity is decreased, as in the slow-wave EEG state like sleep.

### CORTICAL ACTIVITY IS NECESSARY FOR BG ACTIVITY

**FIGURE 2:**
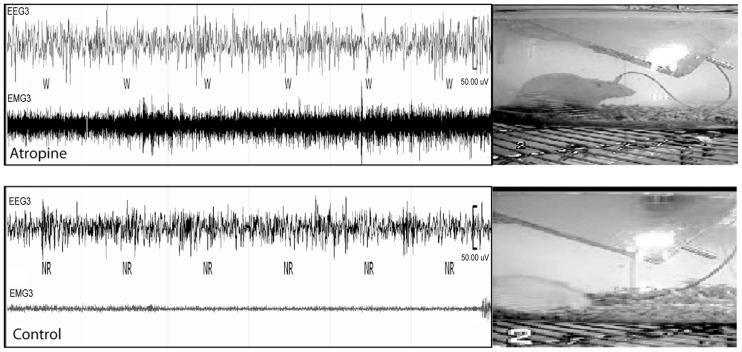
**Atropine induces slow-wave EEG and high EMG.** In 60 s EEG/EMG and video recording trace, a rat shows high EMG, standing posture but sleep-like slow-wave EEG after atropine injection (upper panel), which is very different from normal sleep behavior with slow-wave EEG, low EMG, and sleep posture (lower panel). W = wake, NR = NREM sleep.

**FIGURE 3:**
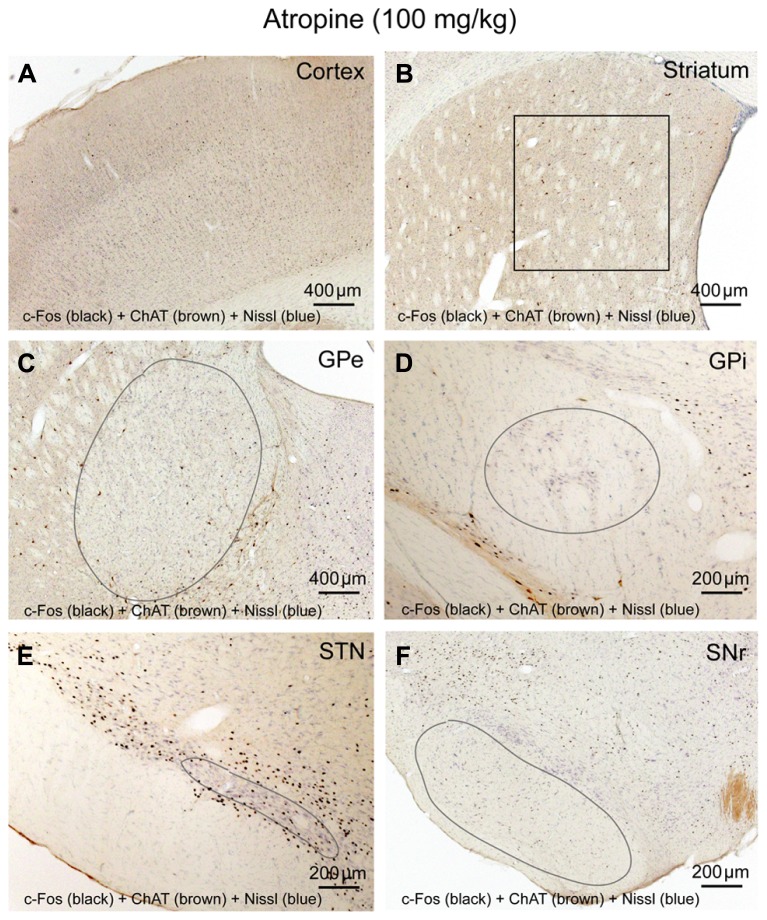
**Atropine silences BG-cortex network.** c-Fos expression in cortex **(A)**, striatum **(B)**, GPe **(C)**, GPi **(D)**, STN **(E)**, and SNr **(F)** after atropine administration is low, despite highly activated sub-cortical arousal systems (see **Figures 6A,C,E,G**).

To determine whether cortical activity is necessary for BG activity, we injected atropine, which induces a unique dissociative behavioral state with slow-wave EEG but heightened locomotion (Irmis, 1971; Davis et al., 2011). This “sleepwalking-like” state provides a unique opportunity to investigate whether cortical activity is necessary for BG activity. We injected atropine (100 mg/kg) at 9:00–10:00 AM and perfused the rats 2 h post-injection. The brains were sectioned, and the sections were immunolabeled with c-Fos and ChAT. Slow-wave EEG shown by atropine administration (Schaul et al., 1978) was confirmed by our EEG/EMG/video recording from two rats (**Figure 2**). Inconsistent with slow EEG, c-Fos expression in the cortex was very low (**Figure 3A**) and comparable to low c-Fos expression in the cortex in sleep condition (**Table 1**). In contrast, c-Fos was highly expressed in sub-cortical arousal regions such as the thalamus (intralaminar and midline region, **Figure 6A**), basal forebrain cholinergic neurons (**Figure 6C**), the tuberomammillary nucleus (TMN; **Figure 6E**), and the locus coeruleus (LC; **Figure 6G**), all resembling the pattern of c-Fos present in these areas during AW (**Table 1**). Along with low cortical activity, and despite high sub-cortical arousal activity, c-Fos expression in all BG subunits, following low cortical c-Fos expression, was low (**Table 1**; **Figures 3B–F**), resembling the sleep condition. These results indicate that despite active subcortical arousal (locomotion) and active subcortical inputs to the BG, which was inactive when cortex was silenced. Thus cortical activity is necessary for BG neuronal activity.

### NMDA RECEPTORS MEDIATE CORTICAL INPUTS TO THE CPu

We hypothesized that the cortex activates BG subunits by activating the striatum and the STN first via direct projections. Cortical input to the BG employs NMDA receptors as part of glutamatergic pathways (Ravenscroft and Brotchie, 2000). As these pathways potentially mediate cortical control over the BG, we examined whether NMDA and non-NMDA receptors are involved in both cortico-striatal and cortico-STN pathways, as well as pathways within the BG.

**FIGURE 4:**
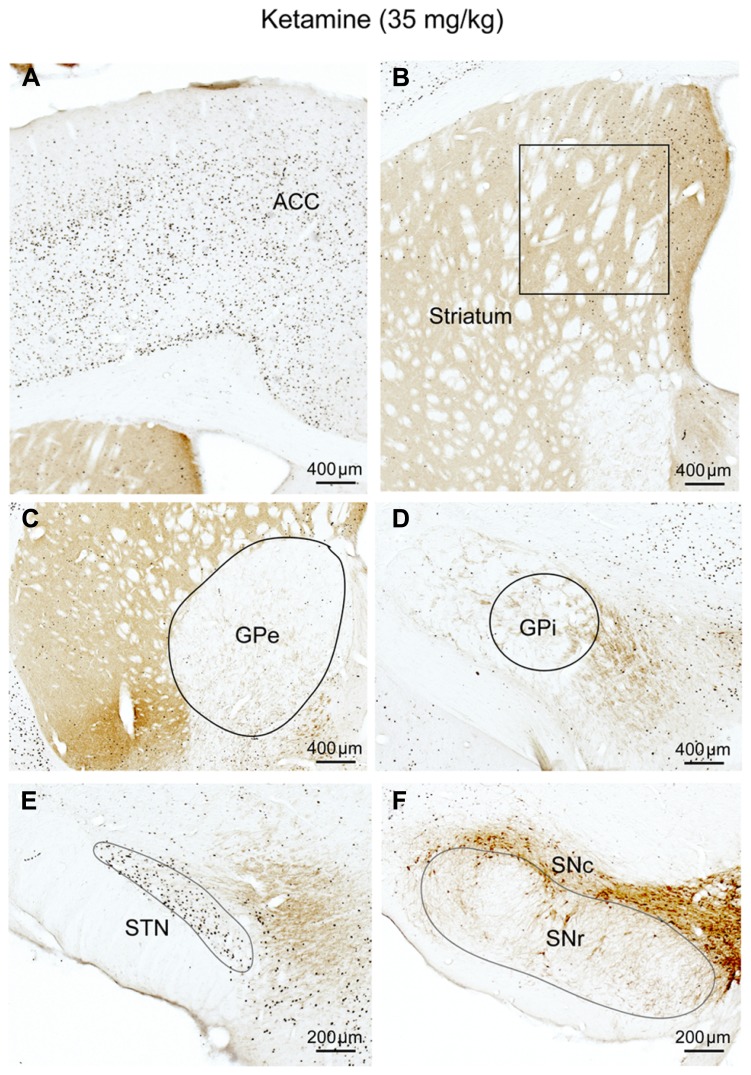
**Ketamine (NMDA receptor antagonist) suppresses corticostriatal inputs.** c-Fos expression in cortex **(A)**, CPu **(B)**, GPe **(C)**, GPi **(D)**, STN **(E)**, and SNr **(F)** after subanesthetic ketamine administration. Despite c-Fos positive neurons are abundant in cortex **(A)**, c-Fos activity in the striatum **(B),** and GPe **(C)** is significantly reduced, compared to AW, but is higher than sleep condition, on the other hand, c-Fos activity in the STN **(E)** is comparable to the AW condition. Fos expression in the GPi, **(D)** and SNr **(F)** is similar to sleep condition (see **Figure 1**; **Table 1**). These results suggest that the NMDA receptors are mostly responsible for cortical control of the striatum and GPe, and entirely responsible for STN control of GPi/SNr.

We administrated ketamine, a NMDA receptor antagonist, at a sub-anesthetic dose (35 mg/kg) to five rats in 9:00–10:00AM. As described before (Lu et al., 2008), animals were awake and active but rarely jumped to the other cages. Animals were perfused 2 h post-injection and the brains were sectioned and immunolabeled with c-Fos and TH. Ketamine at this dose increased locomotor behavior, wake-like EEG, and high c-Fos expression in arousal system structures including orexinergic neurons of the hypothalamus, LC, TMN, cortex, and thalamus, as previously demonstrated (Lu and Greco, 2006; Lu et al., 2008). Despite the increased c-Fos in the cortex, subcortical arousal systems, and thalamus system (**Table 1**; **Figure 4A**), as well as the presence of arousal behavior, c-Fos expression was lower in the CPu, GPi GPe, and SNr than during AW, although c-Fos in the CPu and GPe was higher than during sleep or atropine condition (**Figures 4B,F**; **Table 1**). The STN showed high c-Fos expression, similar to the AW condition (**Table 1**; **Figures 4C–E**). These results suggest that NMDA receptors are involved in mediating cortical glutamate inputs to the CPu and STN glutamate inputs to the GPe, GPi, and SNr but not cortical glutamate inputs to the STN (**Figure 9A**). However, the finding that c-Fos in the CPu and GPe was higher than in atropine and sleep condition suggests non-NMDA receptors may also be involved in inputs to these structures.

### NON-NMDA RECEPTORS MEDIATE CORTICAL INPUTS TO THE STN

As NMDA receptors do not appear to entirely mediate cortical input to the striatum and STN input to the GPe, we examined the role of non-NMDA AMPA/kainate receptors in mediating glutamatergic cortical inputs to the BG. We injected CNQX, an AMPA/kainate receptor antagonist (3.0 mg/kg) at 9:00–10:00 AM in five rats and perfused those 2 h post-injection. The brains were sectioned and immunolabeled with c-Fos.

**FIGURE 5:**
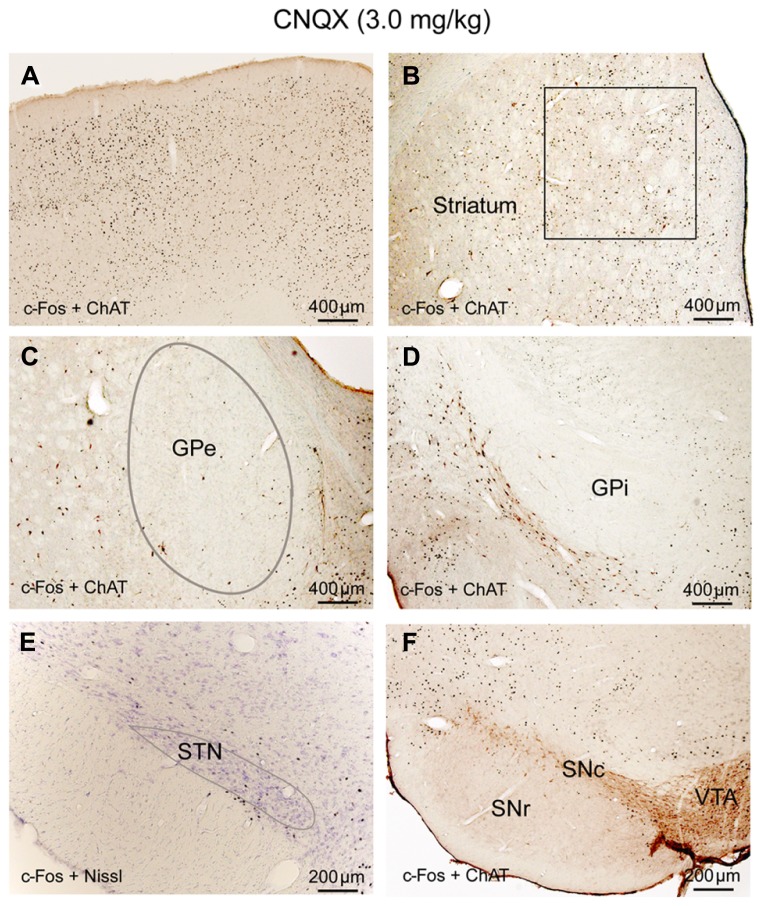
**CNQX (non-NMDA receptor antagonist) suppresses corticosubthalamic inputs.** After CNQX administration, c-Fos is highly expressed in cortex** (A)** and striatum **(B)** while c-Fos in the GPe **(C)**, GPi **(D)**, STN **(E)**, and SNr **(F)** is very low, similar to sleep condition (see **Figure 1**; **Table 1**). Because of Fos expression in the striatum **(B)** is higher than ketamine condition and less than AW, we hypothesize that both NMDA and non-NMDA receptors are involved in mediating cortical input to the striatum, with dominant control from the NMDA receptors. Low Fos activity in the STN indicates that non-NMDA receptors mediate cortical input to the STN. Low Fos in the STN may result in low Fos activity in the GPe, GPi, and SNr.

**FIGURE 6:**
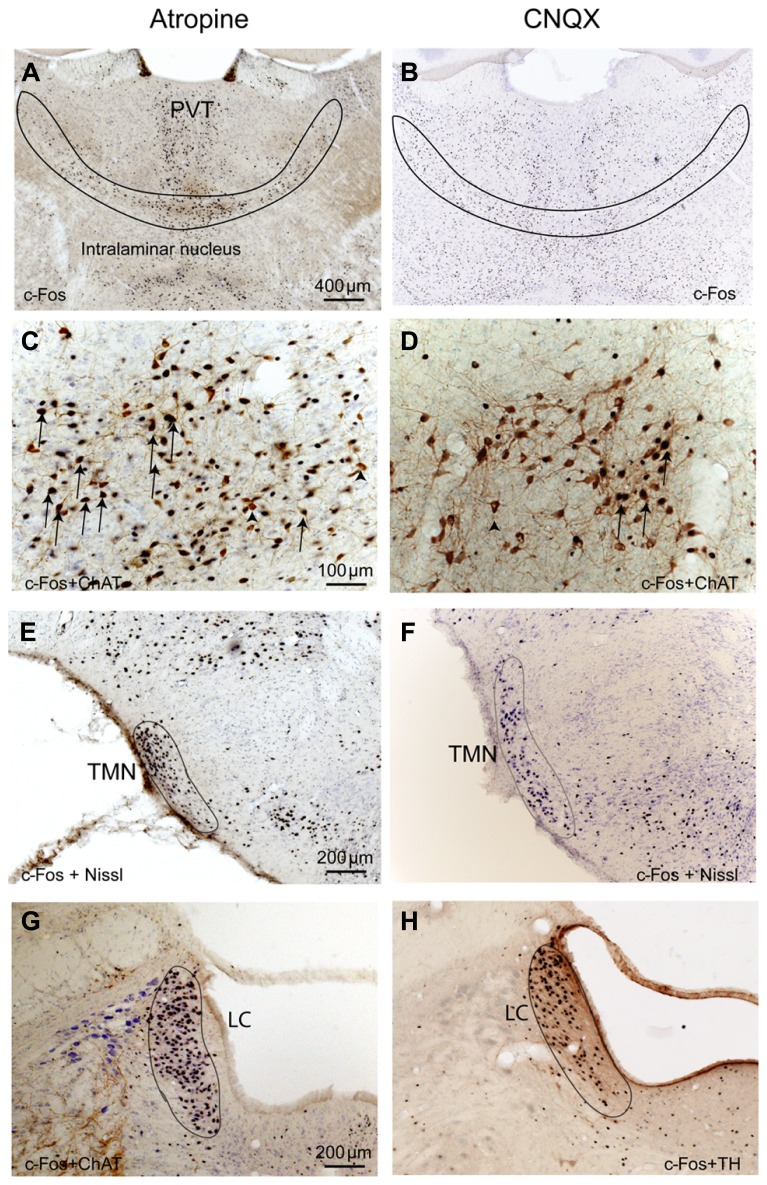
**Atropine and CNQX activate subcortical ascending arousal systems.** After both atropine and CNQX administration, c-Fos stained neurons are present in the thalamus **(A,B)**, basal forebrain cholinergic neurons **(C,D)**, TMN histaminergic neurons **(E,F)** and LC noradrenergic neurons **(G,H)**. Arrows in C and D indicate typical double-labeled neurons containing c-Fos and ChAT.

Until perfusion time, rats with CNQX injections were awake but appeared to have difficulty with locomotion; their body posture suggested low muscle tone (partial paralysis). c-Fos expression in the cortex and thalamus, as well as subcortical arousal systems including the TMN, LC, and BF were comparable to the ketamine and AW condition (**Table 1**; **Figures 5A** and **6B,D,F,H**). c-Fos in the striatum was higher than ketamine injection but less than the AW condition, c-Fos was absent in the GPe, GPi, SNr, and STN (**Table 1**; **Figures 5B–F**). These results, in conjunction with patterns of c-Fos expression during ketamine, suggest that non-NMDA (AMPA/kainate) receptors predominantly mediate cortical input to the STN, and they together with NMDA receptors mediate cortical input to the striatum, and STN input to the GPe (**Figure 9A**).

### DOPAMINE DEPLETION ALTERS BG AND CORTICAL ACTIVITY DURING ACTIVE WAKE

The CPu receives dopaminergic input from the substantia nigra pars compacta (SNc). To determine how this input regulates the BG, we selectively destroyed SNc dopamine efferents to the BG by unilaterally injecting 6-OHDA into the ventral GPe in five rats. This lesion method has also been used and been verified recently in behavioral tests (Abedi et al., 2013). After the rats recovered from surgery, we examined the role of dopamine depletion on cortical input to the BG using two conditions: high cortical activity (2 h of AW starting at 9:00–10:00 AM) and low cortical activity (2 h of sleep starting at 9:00–10:00 AM). After AW or sleep conditions, animals were perfused, and two series of sections were separately labeled with TH and c-Fos.

Tyrosine hydroxylase immunoreactivity showed selective loss of TH terminals in the dorsal striatum (caudate and putamen) of the 6-OHDA-injected animals, indicating dopamine depletion. The ventral striatum (nucleus accumbens and olfactory tubercle) and the dopaminergic neurons and terminals in the hypothalamus and amygdala were intact. In addition, LC TH-labeled neurons were intact.

**FIGURE 7:**
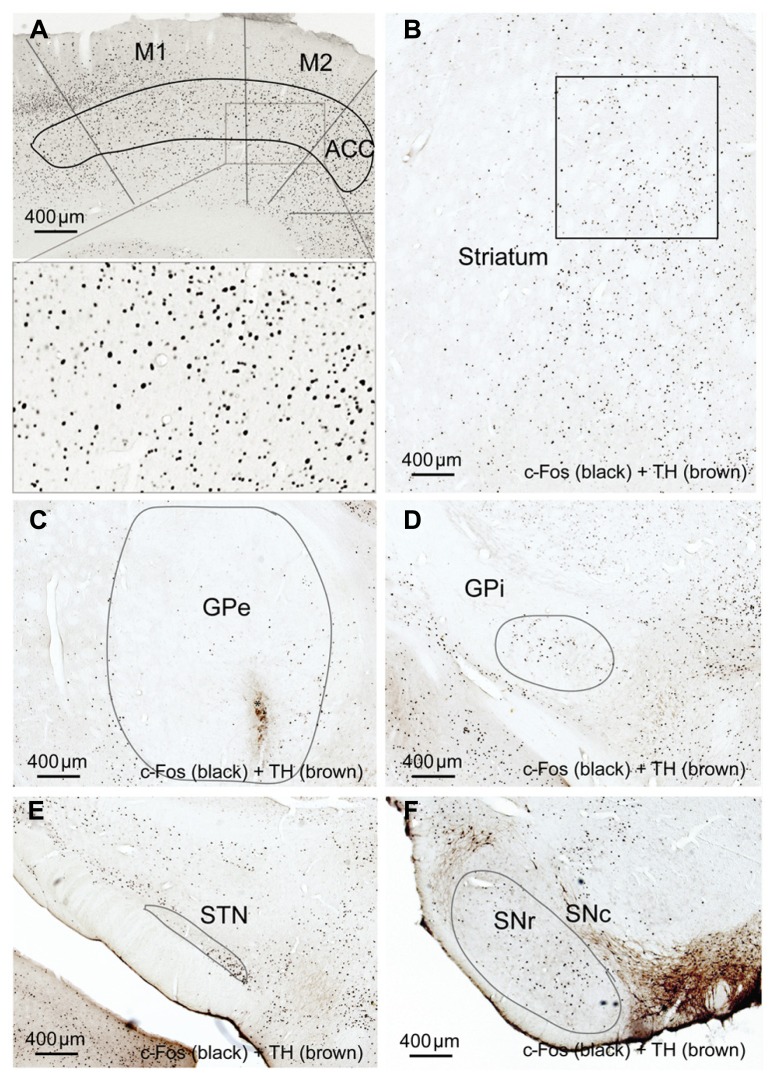
**Striatal dopamine depletion alters activity in cortex-BG.** Compared to the unlesion side, c-Fos expression after dopamine depletion is increased in motor (M1-2) cortex** (A)**, GPi **(D)** and SNr **(F)** but reduced in the striatum **(B)**, GPe, **(C)** and STN **(E)**. M1: primary motor cortex, M2: secondary motor cortex.

**FIGURE 8:**
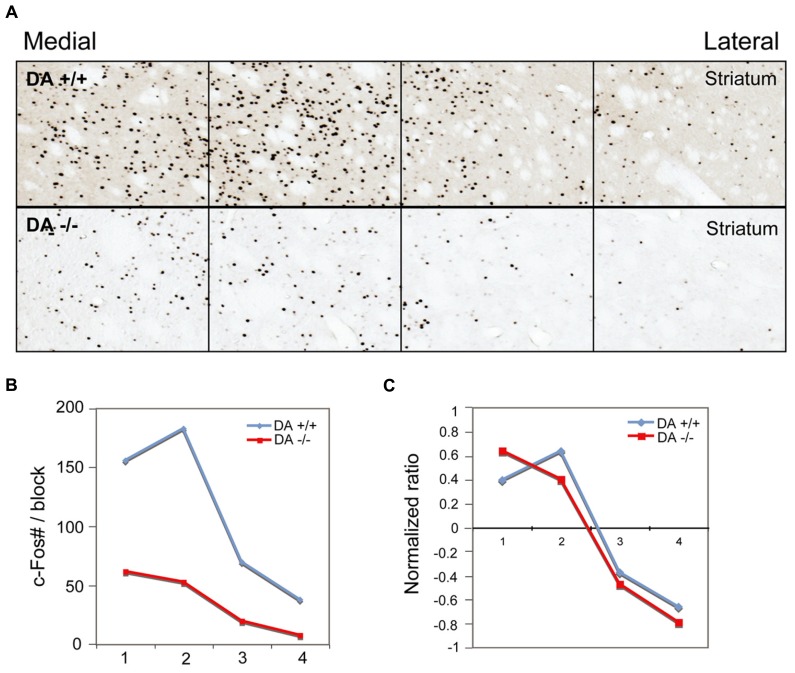
**Dopamine depletion evenly reduces c-Fos expression in the striatum.** After unilateral striatal dopamine depletion, c-Fos expression in the striatum in AW is reduced in the lesion side, compared to the intact side **(A)** c-Fos counts** (B)** and normalized value (count–average)/average **(C)** in four striatal counting areas indicate the reduction of c-Fos positive neurons by 6-OHDA is evenly distributed across the striatum.

In the high cortical activity AW condition, the number of large and intensely c-Fos-stained neurons in motor cortex M1 and M2 putative layer V neurons were higher in the lesioned side than the unlesioned side (**Table 2**; **Figure 7A**). Compared to the unlesioned side, dopamine depletion greatly reduced overall the number of c-Fos stained neurons in the CPu (**Table 2**; **Figure 7B**). c-Fos reduction appeared to be even across the striatum (**Figure 8**). Likewise, the number of c-Fos stained neurons in the GPe and STN was substantially reduced (**Figures 7C,E**). In contrast, the number of c-Fos positive neurons in the GPi and SNr of the lesioned side was twice that of the unlesioned side (**Table 2**; **Figures 7D,F**).

In the low cortical activity sleep condition, we again observed loss of TH terminals in the dorsal striatum, with preserved TH expression in the ventral striatum, hypothalamus, amygdala, and LC. In contrast to the AW condition, in the low cortical activity sleep condition c-Fos expression in the BG in both lesioned and unlesioned sides was uniformly low, without any differences between the sides (data not shown). These results indicate that dopamine depletion alters BG activity during high cortical activity, but not during low cortical activity.

## DISCUSSION

**FIGURE 9:**
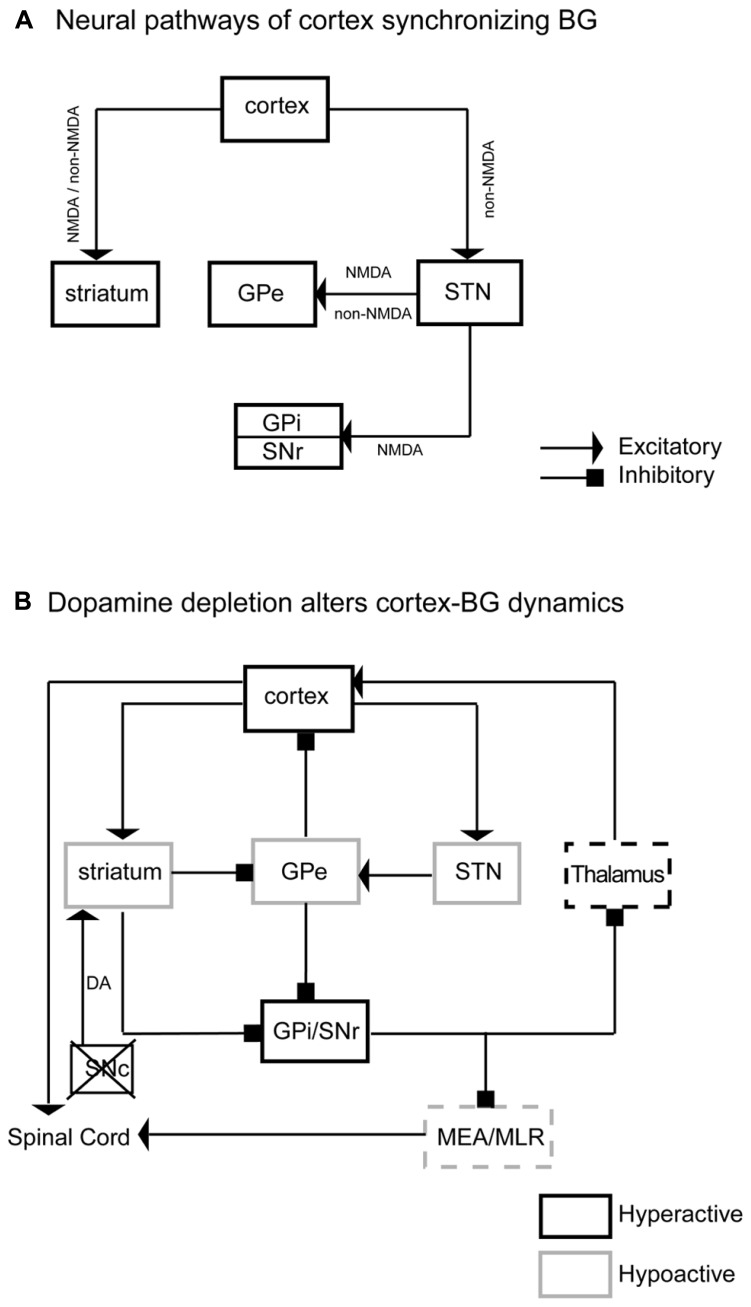
**Neurodynamic model of BG–cortex interactions.** Panel** A **illustrates the neural pathways, neurotransmitters, and receptors by which cortex synchronizes the BG. This neural circuitry regulates BG activity. Panel **B** illustrates specific neuronal activity changes and underlying pathways in the BG network and BG-cortex circuit. DA depletions reduce activity in the striatum, GPe, and STN and increase neuronal activity in the GPi and SNr. Hypoactive GPe causes hyperactive cortex, specifically M1–M2. Hyperactive GPi/SNr may reduce pontine motor control area via the midbrain extrapyramidal area (MEA) and midbrain locomotor region (MLR). The black box indicates a hyperactive region, while light boxes indicate hypoactive regions. Dashed boxes are areas that fail to show expected changes in c-Fos expression based on this model.

We have demonstrated that BG (CPu, GPe, GPi, SNr, and STN) neuronal activity was synchronized to, and driven by, cortical neuronal activity via two routes, corticostriatal and corticosubthalamic projections; the STN then activated the GPe, SNr, and GPi to complete the cortex-BG synchronization (**Figure 9A**). NMDA and non-NMDA receptors mediated cortical glutamatergic inputs to the striatum as well as STN inputs to the GPe; NMDA receptors mediated STN inputs to the SNr and GPi; non-NMDA receptors mediated cortical inputs to the STN (**Figure 9A**). Dopamine depletion reduced activity in the CPu, GPe, and STN but increased activity in the GPi, SNr, and motor cortex (**Figure 9B**). These changes were particularly prominent in the active arousal conditions.

We used c-Fos as a marker of neuronal activity, although there are some limitations with using c-Fos. For example certain neurons such as pontine cholinergic neurons, midbrain dopaminergic neurons and pontine serotonergic neurons do not show robust c-Fos staining (Lu et al., 2006). On the other hand, studies in sleep-wake system, circadian and feeding regulation indicate that c-Fos in other brain regions correlate with neuronal firing activity. Notably, all brain areas in our study are able to express c-Fos, which suggests that c-Fos in those areas reflects neuronal activity. And, importantly, we examined the contrast in c-Fos expression in the same area between conditions.

BG subunits are more active (firing rates) during cortically active states like wake and REM sleep (Magill et al., 2000; Urbain et al., 2000; Lee et al., 2001). Even during sleep, striatal, GPe and STN firing activity all follow up and down states of slow oscillatory cortical activity (Magill et al., 2000; Urbain et al., 2000; Lee et al., 2001; Kasanetz et al., 2002; Mahon et al., 2006). The responses of BG (CPu, GPe, GPi, STN, and SNr) neurons to a single cortical stimulation exhibit a sequential activity pattern of excitation-inhibition-excitation (Kita and Kita, 2011). The first excitation of BG neurons results from direct cortical projections to the striatum and STN, which then stimulates GPi, GPe, and SNr (Tachibana et al., 2008). The subsequent inhibition and excitation are likely mediated by intra-BG interactions (Afsharpour, 1985; Rouzaire-Dubois and Scarnati, 1985). While BG and cortex appear synchronized, it was unclear whether activity of BG subunits depended on active cortical input or if subcortical input was sufficient to drive BG activity. Systemic atropine administration produced a dissociative state of cortical inhibition, subcortical activation, and BG inhibition, suggesting that the cortical inputs, rather than subcortical inputs, are required and necessary to drive overall BG activity. Subcortical inputs to the BG, including midbrain dopaminergic, pontine serotonergic and thalamic glutamatergic projections, may modulate BG activity during cortical active states. These results suggest that the BG activity is driven by cortical activity. Moreover, without active cortical inputs to the BG, such as during sleep or anesthesia, dopamine has a minimal role in modulating BG activity.

Aberrant or diminished cortical activity, such as those observed in the motor cortex in MPTP- treated primates, is thought to produce Parkinsonian symptoms (Goldberg et al., 2002; Johnson et al., 2009; Pasquereau and Turner, 2011). However, 6-OHDA lesions of SNc in rats increase firing rates of the prefrontal pyramidal neurons *in vitro* (Zhang et al., 2011) and *in vivo* (Fan et al., 2011), which is consistent with our observation of high Fos expression in the motor cortex after 6-OHDA lesion. We also observed diminished striatal, GPe, and STN activity, along with hyperactivity in the GPi and SNr with dopamine depletion during active wake. The GPi/SNr hyperactivity may be due to loss of D1 action in the striatonigral neurons and/or hypoactive GPe, while hypoactivity in the STN may be caused by loss of direct dopamine D5 receptor action in the STN neurons (Ni et al., 2001; Baufreton et al., 2003). The BG can access cortex (**Figure 9B**) via the GPe directly (Parent and De Bellefeuille, 1983; Saper, 1984; Haber et al., 1985; Hur and Zaborszky, 2005; Vetrivelan et al., 2010), or indirectly through the mediodorsal thalamic nucleus (MD) (Haber et al., 1985). In addition, the motor thalamus relays SNr/GPi signals to the cortex. Hyperactivity of the GPi/SNr may influence motor cortex via this pathway, resulting in dyskinesia, postural instability, and difficulty turning the body in Parkinsonism. We could not detect c-Fos expression changes in the motor thalamus in the dopamine depletion condition, suggesting that GPi/SNr hyperactivity produces a subtle alteration of firing patterns rather than complete silencing of activity to influence thalamocortical projections.

In summary, our data supports the notion that cortical activity but not subcortical activity is required for BG activation. Specific glutamate receptors are involved in cortical control of the BG, as well as the interaction of BG subunits and ultimately BG output back to cortex. By regulating BG activity during active states, dopamine influences cortical activity to affect sleep-wake and motor behavior.

## Conflict of Interest Statement

The authors declare that the research was conducted in the absence of any commercial or financial relationships that could be construed as a potential conflict of interest.

## References

[B1] Abedi P. M. Delaville C. De Deurwaerdere P. Benjelloun W. Benazzouz A. (2013). Intrapallidal administration of 6-hydroxydopamine mimics in large part the electrophysiological and behavioral consequences of major dopamine depletion in the rat. *Neuroscience* 236 289–297 10.1016/j.neuroscience.2013.01.043 23376117

[B2] Afsharpour S. (1985). Topographical projections of the cerebral cortex to the subthalamic nucleus. *J. Comp. Neurol.* 236 14–28 10.1002/cne.902360103 2414329

[B3] Baufreton J. Garret M. Rivera A. De La Calle A. Gonon F. Dufy B. (2003). D5 (not D1) dopamine receptors potentiate burst-firing in neurons of the subthalamic nucleus by modulating an L-type calcium conductance. *J. Neurosci.* 23 816–825 12574410 10.1523/JNEUROSCI.23-03-00816.2003 PMC6741933

[B4] Davis C. J. Clinton J. M. Jewett K. A. Zielinski M. R. Krueger J. M. (2011). Delta wave power: an independent sleep phenotype or epiphenomenon? *J. Clin. Sleep Med.* 7 S16–S18 10.5664/JCSM.1346 22003323 PMC3190419

[B5] Detari L. Juhasz G. Kukorelli T. (1987). Neuronal firing in the pallidal region: firing patterns during sleep-wakefulness cycle in cats. *Electroencephalogr. Clin. Neurophysiol.* 67 159–166 10.1016/0013-4694(87)90039-3 2439293

[B6] Fan L. L. Zhang Q. J. Liu J. Feng J. Gui Z. H. Ali U. (2011). In vivo effect of 5-HT(7) receptor agonist on pyramidal neurons in medial frontal cortex of normal and 6-hydroxydopamine-lesioned rats: an electrophysiological study. *Neuroscience* 190 328–338 10.1016/j.neuroscience.2011.06.011 21684321

[B7] Goldberg J. A. Boraud T. Maraton S. Haber S. N. Vaadia E. Bergman H. (2002). Enhanced synchrony among primary motor cortex neurons in the 1-methyl-4-phenyl-1,2,3,6-tetrahydropyridine primate model of Parkinson’s disease. *J. Neurosci.* 22 4639–4653 12040070 10.1523/JNEUROSCI.22-11-04639.2002 PMC6758785

[B8] Gompf H. S. Mathai C. Fuller P. M. Wood D. A. Pedersen N. P. Saper C. B. (2010). Locus ceruleus and anterior cingulate cortex sustain wakefulness in a novel environment. *J. Neurosci.* 30 14543–14551 10.1523/JNEUROSCI.3037-10.2010 20980612 PMC2989851

[B9] Haber S. N. Groenewegen H. J. Grove E. A. Nauta W. J. (1985). Efferent connections of the ventral pallidum: evidence of a dual striato pallidofugal pathway. *J. Comp. Neurol.* 235 322–335 10.1002/cne.902350304 3998213

[B10] Hur E. E. Zaborszky L. (2005). Vglut2 afferents to the medial prefrontal and primary somatosensory cortices: a combined retrograde tracing in situ hybridization study [corrected]. *J. Comp. Neurol.* 483 351–373 10.1002/cne.20444 15682395

[B11] Irmis F. (1971). Dissociation between EEG and spontaneous behaviour of rats after atropine. *Act. Nerv. Super. (Praha)* 13 217–218 5113845

[B12] Johnson M. D. Vitek J. L. Mcintyre C. C. (2009). Pallidal stimulation that improves parkinsonian motor symptoms also modulates neuronal firing patterns in primary motor cortex in the MPTP-treated monkey. *Exp. Neurol.* 219 359–362 10.1016/j.expneurol.2009.04.022 19409895 PMC2730829

[B13] Kasanetz F. Riquelme L. A. Murer M. G. (2002). Disruption of the two-state membrane potential of striatal neurones during cortical desynchronisation in anaesthetised rats. *J. Physiol.* 543 577–589 10.1113/jphysiol.2002.0024786 12205191 PMC2290508

[B14] Kita H. Kita T. (2011). Cortical stimulation evokes abnormal responses in the dopamine-depleted rat basal ganglia. *J. Neurosci.* 31 10311–10322 10.1523/JNEUROSCI.0915-11.2011 21753008 PMC3138188

[B15] Lee R. S. Steffensen S. C. Henriksen S. J. (2001). Discharge profiles of ventral tegmental area GABA neurons during movement, anesthesia, and the sleep-wake cycle. *J. Neurosci.* 21 1757–1766 11222665 10.1523/JNEUROSCI.21-05-01757.2001 PMC6762953

[B16] Lu J. Greco M. A. (2006). Sleep circuitry and the hypnotic mechanism of GABAA drugs. *J. Clin. Sleep Med.* 2 S19–S26 17557503

[B17] Lu J. Jhou T. C. Saper C. B. (2006). Identification of wake-active dopaminergic neurons in the ventral periaqueductal gray matter. *J. Neurosci.* 26 193–202 10.1523/JNEUROSCI.2244-05.2006 16399687 PMC6674316

[B18] Lu J. Nelson L. E. Franks N. Maze M. Chamberlin N. L. Saper C. B. (2008). Role of endogenous sleep-wake and analgesic systems in anesthesia. *J. Comp. Neurol.* 508 648–662 10.1002/cne.21685 18383504 PMC4924624

[B19] Magill P. J. Bolam J. P. Bevan M. D. (2000). Relationship of activity in the subthalamic nucleus-globus pallidus network to cortical electroencephalogram. *J. Neurosci.* 20 820–833 10632612 10.1523/JNEUROSCI.20-02-00820.2000 PMC6772398

[B20] Mahon S. Vautrelle N. Pezard L. Slaght S. J. Deniau J. M. Chouvet G. (2006). Distinct patterns of striatal medium spiny neuron activity during the natural sleep-wake cycle. *J. Neurosci.* 26 12587–12595 10.1523/JNEUROSCI.3987-06.2006 17135420 PMC6674897

[B21] Nauta H. J. (1979). Projections of the pallidal complex: an autoradiographic study in the cat. *Neuroscience* 4 1853–1873 10.1016/0306-4522(79)90060-5 530436

[B22] Ni Z. Gao D. Bouali-Benazzouz R. Benabid A. L. Benazzouz A. (2001). Effect of microiontophoretic application of dopamine on subthalamic nucleus neuronal activity in normal rats and in rats with unilateral lesion of the nigrostriatal pathway. *Eur. J. Neurosci.* 14 373–381 10.1046/j.0953-816x.2001.01644.x 11553287

[B23] Parent A De Bellefeuille L. (1983). The pallidointralaminar and pallidonigral projections in primate as studied by retrograde double-labeling method. *Brain Res.* 278 11–27 10.1016/0006-8993(83)90222-6 6315152

[B24] Pasquereau B. Turner R. S. (2011). Primary motor cortex of the parkinsonian monkey: differential effects on the spontaneous activity of pyramidal tract-type neurons. *Cereb. Cortex* 21 1362–1378 10.1093/cercor/bhq217 21045003 PMC3097989

[B25] Qiu M. H. Vetrivelan R. Fuller P. M. Lu J. (2010). Basal ganglia control of sleep-wake behavior and cortical activation. *Eur. J. Neurosci.* 31 499–507 10.1111/j.1460-9568.2009.07062.x 20105243 PMC3928571

[B26] Ravenscroft P. Brotchie J. (2000). NMDA receptors in the basal ganglia. *J. Anat.* 196 577–585 10.1046/j.1469-7580.2000.19640577.x 10923988 PMC1468098

[B27] Rouzaire-Dubois B. Scarnati E. (1985). Bilateral corticosubthalamic nucleus projections: an electrophysiological study in rats with chronic cerebral lesions. *Neuroscience* 15 69–79 10.1016/0306-4522(85)90124-1 4010936

[B28] Saper C. B. (1984). Organization of cerebral cortical afferent systems in the rat. II. Magnocellular basal nucleus.* J. Comp. Neurol.* 222 313–342 10.1002/cne.902220302 6699210

[B29] Schaul N. Gloor P. Ball G. Gotman J. (1978). The electromicrophysiology of delta waves induced by systemic atropine. *Brain Res.* 143 475–486 10.1016/0006-8993(78)90358-X 647373

[B30] Tachibana Y. Kita H. Chiken S. Takada M. Nambu A. (2008). Motor cortical control of internal pallidal activity through glutamatergic and GABAergic inputs in awake monkeys. *Eur. J. Neurosci.* 27 238–253 10.1111/j.1460-9568.2007.05990.x 18093168

[B31] Urbain N. Gervasoni D. Souliere F. Lobo L. Rentero N. Windels F. (2000). Unrelated course of subthalamic nucleus and globus pallidus neuronal activities across vigilance states in the rat. *Eur. J. Neurosci.* 12 3361–3374 10.1046/j.1460-9568.2000.00199.x 10998119

[B32] Vetrivelan R. Fuller P. M. Tong Q. Lu J. (2009). Medullary circuitry regulating rapid eye movement sleep and motor atonia. *J. Neurosci.* 29 9361–9369 10.1523/JNEUROSCI.0737-09.2009 19625526 PMC2758912

[B33] Vetrivelan R. Qiu M. H. Chang C. Lu J. (2010). Role of Basal Ganglia in sleep-wake regulation: neural circuitry and clinical significance. *Front. Neuroanat.* 4:145 10.3389/fnana.2010.00145 PMC2996256 21151379

[B34] Yelnik J. (2008). Modeling the organization of the basal ganglia. *Rev. Neurol. (Paris)* 164 969–976 10.1016/j.neurol.2008.04.019 18808769

[B35] Zhang Q. J. Li L. B. Niu X. L. Liu J. Gui Z. H. Feng J. J. (2011). The pyramidal neurons in the medial prefrontal cortex show decreased response to 5-hydroxytryptamine-3 receptor stimulation in a rodent model of Parkinson’s disease. *Brain Res.* 1384 69–79 10.1016/j.brainres.2011.01.086 21291871

